# The neutrophil-to-lymphocyte ratio in Parkinson's disease: a systematic review and meta-analysis of its association with differential diagnosis, severity, and prognosis

**DOI:** 10.1016/j.prdoa.2026.100494

**Published:** 2026-08-15

**Authors:** Miguel Cabanillas-Lazo, Alvaro Montes-Baldarrago, Sandra Uriol-Alvino, Hans Baltazar-Ñahui, Natalia B. Pereda-Leon, Harold Jimenez-Alvarez, Frank Mayta-Tovalino

**Affiliations:** a Grupo de Investigación NEMECS, Neurociencias, Metabolismo, Efectividad Clínica y Sanitaria, Universidad Científica del Sur, Lima, Peru; b Sociedad Cientifica de San Fernando, Lima, Peru; c Facultad de Medicina de San Fernando, Universidad Nacional Mayor de San Marcos, Lima, Peru; d Universidad Nacional del Santa, Peru; e Sociedad Científica de Estudiantes de Medicina de la Universidad Nacional de Trujillo, Trujillo, Peru; f Vicerrectorado de Investigación, Universidad San Ignacio de Loyola, Lima, Peru

**Keywords:** Parkinson's disease, Neutrophil-to-lymphocyte ratio, Biomarker, Neuroinflammation

## Abstract

**Background and aim:**

This study aimed to systematically review and meta-analyze the evidence on the association of the neutrophil-to-lymphocyte ratio (NLR) with clinical parameters in Parkinson's disease (PD), specifically its role in group differentiation (from healthy controls and other neurodegenerative diseases), disease severity, and prognosis.

**Methods:**

In this study, a systematic review and meta-analysis were performed according to the PRISMA guidelines. A search conducted in PubMed, Embase, Scopus, Web of Science, and Google Scholar up to June 2024 was undertaken, providing observational studies that reported NLR in PD. The random-effects models pooled the mean differences of PD versus controls and PD versus multiple system atrophy (MSA), and pooled for UPDRS subscale correlation coefficients, total UPDRS, and Hoehn & Yahr (H&Y) stage. Constipation, depressive symptomatology, and mild cognitive impairment (MCI) markers were also analyzed.

**Results:**

Twenty-eight studies were included. The NLR was significantly higher in patients with PD than in healthy controls (MD, 0.38; 95% CI 0.22–0.53; I^2^ = 90%; moderate certainty). No significant difference was observed between PD and MSA. NLR showed moderate positive correlations with UPDRS I (*r* = 0.42), UPDRS II (*r* = 0.34), UPDRS III (*r* = 0.27), total UPDRS (*r* = 0.67), and H&Y stage (*r* = 0.33). Associations were significant for depressive symptoms (high certainty) but not for constipation. For MCI, the NLR was lower rather than higher in patients who developed impairment, with modest discriminatory performance (moderate certainty).

**Conclusion:**

NLR is higher in PD and correlates with clinical severity and depressive symptoms, but it does not discriminate PD from MSA or between motor subtypes, and it was not associated with incident mild cognitive impairment in the expected direction.

## 1 Introduction

Parkinson's disease (PD) is a neurodegenerative movement disorder characterized by rigidity, bradykinesia, and tremor [1]. As of 2016, it had affected 6.1 million people and caused 211,296 deaths, up from 2.5 million reported in 1990. In addition, 3.2 million disability-adjusted life years, a measure that includes years lived with disability and years lost due to the disease, were recorded in the same year [2]. These increases are linked to population growth and aging, genetic predisposition, lifestyle changes, and environmental pollution [3]. The symptomatology of PD triggers progressive disability, reduced quality of life, and high economic costs for countries [4], [5]. In addition, patients with PD have an increased risk of all-cause mortality [6].

Two features are central to the pathophysiology of PD: degeneration of substantia nigra neurons and the presence of Lewy bodies [7]. The presence of these stimuli triggers the expression of the neurotoxic phenotype of microglia, which produce cytokines such as TNFα, IL-6, and IL-1b, in addition to proinflammatory chemokines that contribute to neuroinflammation, triggering degeneration of the SN and affecting the integrity of the BBB [8]. Impairment of the blood-brain barrier allows lymphocytes (CD4+ and CD8+) to pass from the peripheral circulation to sites of neuronal damage in the brain [9]. Inflammation is not confined to the brain. Immune dysregulation of the nasal or intestinal mucosa triggers an increase in peripheral inflammation, which contributes to dopaminergic dysfunction [10]. Proinflammatory cytokines induce the synthesis of chemokines that cause neutrophil and monocyte recruitment into the peripheral blood [11]. Beyond microglial activation, PD involves both arms of the immune system. Peripheral monocytes display a primed, pro-inflammatory profile, and adaptive responses to alpha-synuclein derived antigens have been described alongside impaired regulatory T cell function, which together shift the peripheral compartment towards a neutrophil-predominant, relatively lymphopenic state [9], [10]. It is this bidirectional imbalance, rather than neutrophilia or lymphopenia in isolation, that ratios derived from the complete blood count attempt to capture, and it is shared, to a variable extent, with other neurodegenerative and atypical parkinsonian conditions.

In daily practice, the diagnosis of PD is based on clinical criteria, accompanied by imaging tests to rule out the disease [12]. An autopsy is a diagnostic confirmation [13]. However, low diagnostic accuracy has been reported in the early stages of the disease [14]. Additionally, so far, no accessible biomarkers for this synucleinopathy have been identified [15], [16]. Multiple predictors associated with disease progression have been searched for prognosis; however, more studies are required for their validation [17]. Therefore, it is necessary to search for useful markers in the disease diagnosis and prognosis.

The neutrophil-to-lymphocyte ratio (NLR) was originally proposed in the intensive care setting as a rapid, inexpensive index of systemic inflammation and physiological stress, and its clinical relevance was subsequently consolidated in cardiovascular and cerebrovascular disease, where it predicts both events and outcome [18], [19], [20]. It is derived from the routine complete blood count and integrates two opposing arms of the immune response: innate activation, expressed as neutrophilia driven in part by endogenous cortisol and catecholamines, and adaptive suppression, expressed as relative lymphopenia. This dual behaviour explains both its appeal and its principal weakness. The NLR is an entirely non-specific parameter and is modified by intercurrent infection, cardiovascular and metabolic comorbidity, smoking, immunomodulatory treatment, acute stress, and even the circadian timing of venepuncture [19]. Within neurology, a high NLR predicts poor prognosis in stroke [21] and is increased in mild cognitive impairment and traumatic brain injury [22]. The same family of complete blood count derived indices, including the platelet-to-lymphocyte ratio (PLR), the lymphocyte-to-monocyte and neutrophil-to-monocyte ratios, the monocyte-to-high-density lipoprotein ratio (MHR), the neutrophil-to-high-density lipoprotein ratio (NHR) and the neutrophil-to-haemoglobin ratio (N/HGBR), has been examined in PD and, increasingly, in atypical parkinsonian syndromes [23], [24], [25], [26].

A previous systematic review confirmed higher NLR values in PD than in healthy controls, but was restricted to that comparison and did not grade the certainty of the evidence [27]. The present systematic review and meta-analysis extends this work by pooling correlations between the NLR and validated severity scales, synthesising non-motor and prognostic outcomes, exploring the sources of the reported heterogeneity, and applying GRADE to every outcome.

## 2 Methods

### 2.1 Protocol and guidelines

This systematic review was performed according to the Preferred Reporting Items for Systematic Reviews and Meta-Analyses (PRISMA) [28]. The study protocol was registered in PROSPERO under the ID CRD42023421902.

### 2.2 Eligibility criteria

Studies were included if they met the following criteria: 1) studies including patients with PD; 2) NLR values to assess diagnosis, severity (exposition and outcome assessed simultaneously) or prognosis (follow-up to measure the outcome); and 3) analytical observational studies (cross-sectional, case-control, and cohort studies). We excluded narrative and systematic reviews, non-human studies, case reports, conference abstracts, and letters.

### 2.3 Search of relevant studies

A systematic search was conducted in PubMed, Embase, Scopus, Web of Science, and Google Scholar until June 2024. The search strategy was tailored for application in additional databases. The search was conducted without imposing language or publication date restrictions. We also reviewed the bibliographic references of the included studies and selected articles that met the specified criteria to ensure comprehensiveness (Supplementary material 1).

### 2.4 Outcomes

This review evaluated the association of the NLR with the following clinical parameters in PD:

- Group differentiation: NLR levels between patients with PD and healthy controls and between patients with PD and those with multiple system atrophy (MSA).
- Motor subtypes: NLR differences between akinetic-rigid and tremor-dominant PD subtypes.
- Disease severity and prognosis: Correlation of NLR with motor symptoms (UPDRS Part III), activities of daily living (UPDRS Part II), non-motor symptoms (UPDRS Part I), global disease severity (UPDRS total score), disease stage (Hoehn and Yahr scale), and non-motor manifestations (association of NLR with constipation, depressive symptoms, and mild cognitive impairment [MCI]) was assessed.

### 2.5 Data extraction

Two authors independently performed data extraction using a data extraction form and mutually resolved disagreements and, ultimately, with a third author. We extracted data on the following: title of study; authors; year of publication; study design; country the sample was assessed in; number of participants; sex; age; sample time; subgroups in PD; mean or median NLR; follow-up crude and adjusted association measures; type and definition of outcome. Where further information was needed, the corresponding author was contacted via email.

### 2.6 Assessment of risk of bias

Two authors independently graded the quality of cohort and case-control studies using the NOS [29]. This scale assesses the quality of published non-randomized studies on the basis of three dimensions: selection, comparability, and outcome/exposure, with a star system applied for each sub-item. Studies with ≥6 stars are said to have a low risk of bias (high quality), while a score of 4–5 stars indicated moderate risk of bias, and < 4 stars a high risk of bias.

### 2.7 Statistical analysis

A meta-analysis was planned for each outcome; however, when this was not possible due to unavailable data, a narrative synthesis was performed: “a narrative overview of the literature with summary counts.” Meta-analyses were performed using the inverse variance method and a random-effects model. The variance between studies (τ2) was estimated using the DerSimonian-Laird method. The mean differences (MD) with their 95% confidence intervals (CI, 95%) were pooled. Pooled correlation coefficients with 95% confidence intervals (CIs) were calculated using the inverse variance weighting method and the restricted maximum likelihood method estimator for between-study variance. Before the analysis, the correlation coefficients were transformed into Fisher's z values. This transformation was performed automatically by the metafor function with the m argument set to “ZCOR”. Heterogeneity between studies was assessed using the I2 statistic. Heterogeneity was defined as low if I2 < 30%, moderate if I2 = 30–60%, and high if I2 > 60%. The metacont function of the meta-package in R 4.1.0 was used (https://www.r-project.org). Subgroup analyses were performed by study design, case-control matching, and geographic region. Finally, a leave-one-out sensitivity analysis was performed.

### 2.8 Assessment of evidence certainty

The certainty of our pooled results and qualitative synthesis was independently rated using the Grading of Recommendation, Assessment, Development, and Evaluation (GRADE) on continuous outcomes, prognostic factor, and test accuracy [30], [31]. This is based on GRADE domains: study limitations (risk of bias of the included studies), imprecision (sample size and confidence interval), indirectness (generalizability), inconsistency (statistical heterogeneity), publication bias, large magnitude of an effect, dose-response gradient, and effect of plausible residual confounding per GRADE handbook. We have adapted the GRADE criteria to our results. The certainty of the evidence was depicted as high, moderate, low, or very low (Table 1).

**Table 1:** Summary of findings.

Outcome	№ of participants (studies)	Certainty of the evidence (GRADE)	Anticipated absolute effects	
			Mean difference (MD)	95% CI
Differential diagnosis				
Difference between PD patients and healthy participants	3593 (22 observational studies)	⨁⨁⨁◯ Moderate^a^	0.38	0.22 to 0.53
Difference between PD vs. Multiple System Atrophy (MSA)	223 (2 observational studies)	⨁⨁⨁◯ Moderate^b^	−0.30	−1.52 to 0.93
Difference between Akinetic-rigid vs. tremor-dominant subtype	124 (2 observational studies)	⨁⨁◯◯ Low^a^, ^b^	0.33	−0.62 to 1.27

Severity outcomes				
Correlation between NLR and UPDRS I	644 (2 observational studies)	⨁⨁⨁◯ Moderate^c^	The studies showed a positive correlation between NLR and UPDRS I (r = 0.42; 95% CI: 0.33 to 0.51)	
Correlation between NLR and UPDRS II	644 (2 observational studies)	⨁⨁⨁◯ Moderate^c^	The studies showed a positive correlation between NLR and UPDRS II (r = 0.34; 95% CI: 0.24 to 0.44)	
Correlation between NLR and UPDRS III	1320 (4 observational studies)	⨁⨁⨁⨁ High	The studies showed a positive correlation between NLR and UPDRS III (r = 0.27; 95% CI: 0.16 to 0.38)	
Correlation between NLR and UPDRS Total Score	644 (2 observational studies)	⨁⨁⨁◯ Moderate^c^	The studies showed a positive correlation between NLR and UPDRS Total Score (r = 0.67; 95% CI: 0.07–0.92)	
Correlation between NLR and Hoehn and Yahr Score	480 (3 observational studies)	⨁⨁⨁◯ Moderate^c^	The studies showed a positive correlation between NLR and Hoehn and Yahr Score (r = 0.33; 95% CI: 0.24 to 0.41)	
Constipation	123 (1 observational study)	⨁⨁⨁◯ Moderate^b^	0.2	−0.4 to 0.8
Depression symptoms	465 (1 observational study)	⨁⨁⨁⨁ High	0.6	0.24 to 0.96

Prognostic outcomes				
Sensitivity for prognosis Mild cognitive impairment (Follow: 3.15 years)	58 (1 observational study)	⨁⨁⨁◯ Moderate^d^	The study showed a sensitivity of 69.6% (CI: 49.13 to 84.4) for prognosis in Mild cognitive impairment	
Specificity for prognosis Mild cognitive impairment (Follow: 3.15 years)	58 (1 observational study)	⨁⨁⨁◯ Moderate^d^	The study showed a specificity of 67.8% (CI: 49.34 to 82.07) for prognosis in Mild cognitive impairment	

Explanations

a High inconsistency was detected in meta-analyses. The calculated I^2^ was >60%.

b Confidence interval crosses the point of no effect

c Imprecise confidence interval (Spearman's rho <0.39)

d Imprecision due to confidence interval less than 70% in one study

## 3 Results

A total of 909 records were identified, of which 360 duplicates were removed. A total of.

**Fig. 1:**
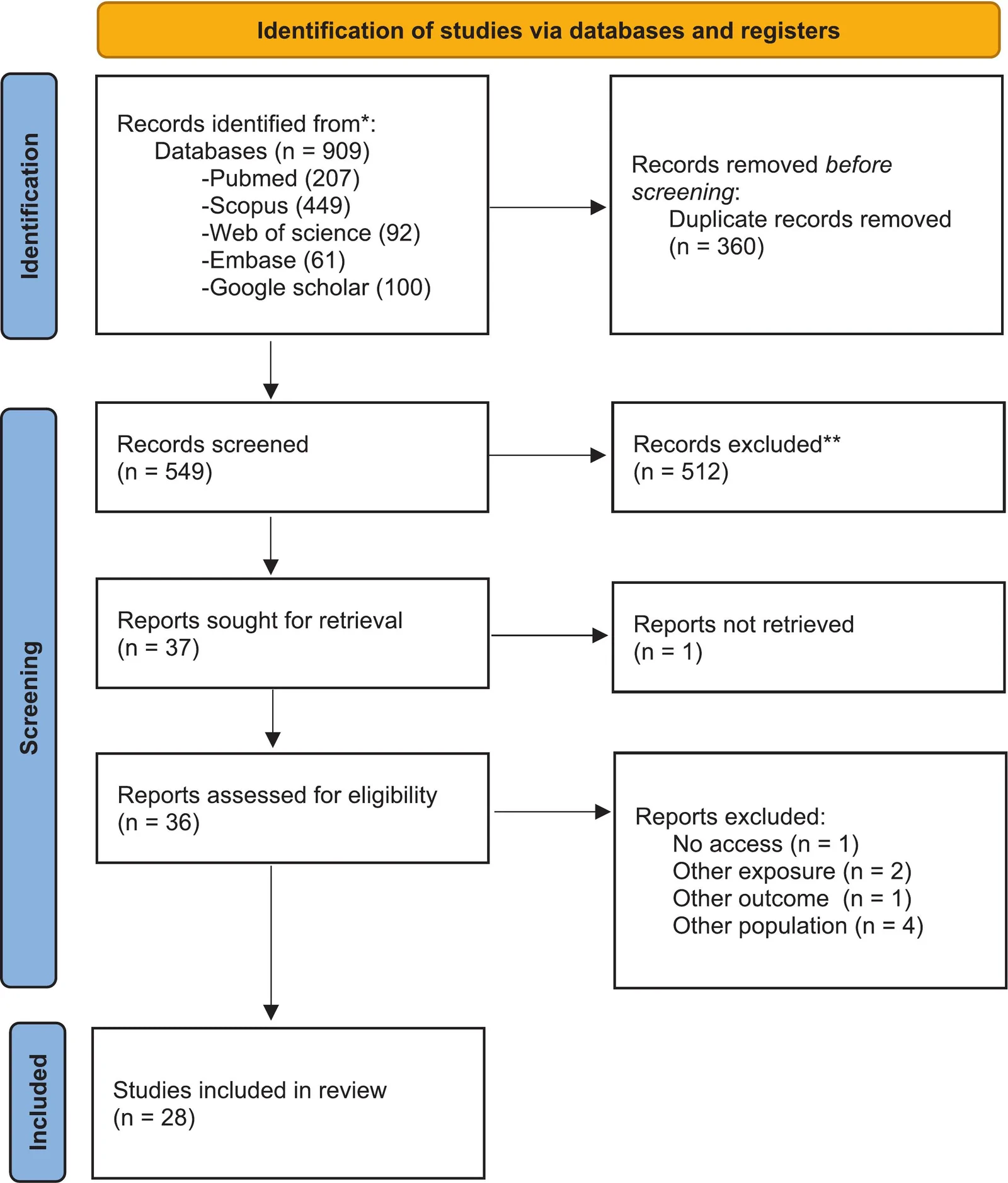
PRISMA Flow diagram.

549 records were screened, and 512 were excluded. Thirty-seven full-text reports were sought for retrieval, one could not be obtained, and 36 were assessed for eligibility. Eight reports were excluded because of no access (*n* = 1), a different exposure (*n* = 2), a different outcome (n = 1) or a different population (*n* = 4) (Supplementary material 2). Ultimately, 28 studies fulfilled the inclusion criteria and were included in the review. The study selection process is illustrated in the PRISMA flow diagram in Fig. 1 [23], [24], [32], [33], [34], [35], [36], [37], [38], [39], [40], [41], [42], [43], [44], [45], [46], [47], [48], [49], [50], [51], [52], [53], [54], [55], [56], [57].

### 3.1 Characteristics of the studies included

The included studies were case-control and cohort studies. Twenty-two studies, comprising 3593 participants, contributed to the pooled comparison of the NLR between patients with PD and healthy controls; the remaining studies contributed to the severity, non-motor and prognostic outcomes. An overview of the characteristics of the included studies is summarized in Supplementary Material 3.

### 3.2 Assessment of risk of bias

The risk of bias of the included studies was evaluated using the NOS. Scores ranged from 6 to 8, with four studies obtaining a maximum score of 8. Therefore, all studies were classified as having a low risk of bias (NOS Supplementary Material 4).

### 3.3 Patients with PD versus healthy controls

**Fig. 2:**
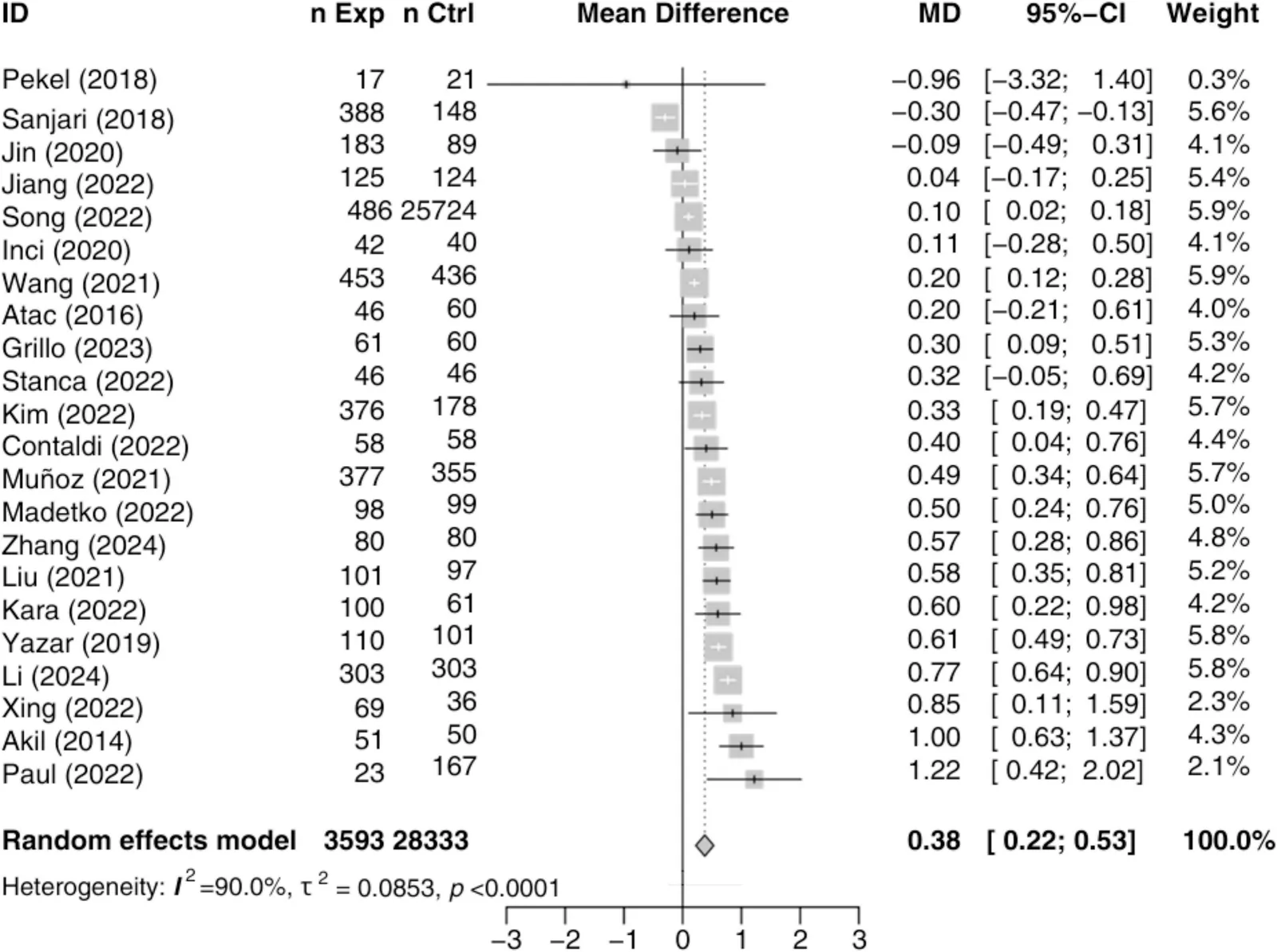
Neutrophil-to-lymphocyte ratio (NLR) values between patients with Parkinson disease and healthy controls.

The pooled results using a random-effects model showed that NLR values were significantly higher in patients with PD than in healthy controls (MD = 0.38; 95% CI: 0.22–0.53) (Fig. 2). The heterogeneity among studies was high (I^2^ = 90.0%).

#### 3.3.1 Subgroup analysis

**According to the study design**

When stratified by study design, retrospective studies showed a significantly higher NLR in PD than in controls (MD = 0.42; 95% CI: 0.25 to 0.59), whereas prospective studies did not (MD = 0.16; 95% CI: −0.31 to 0.62). The test for subgroup differences was statistically significant (*p* = 0.04) (Supplementary material 5).

**According to case-control matching**

When comparing matched and unmatched case–control designs, no significant difference was found between the subgroups. However, unmatched studies exhibited wider CIs crossing zero, indicating greater variability and uncertainty (Supplementary material 6).

**According to the geographic region**

When stratified by continent, the NLR was significantly higher in patients with PD from Asia (MD = 0.37; 95% CI: 0.16 to 0.57) and Europe (MD = 0.44; 95% CI: 0.33 to 0.55).

In contrast, the results from North America did not reach statistical significance (MD = 0.52; 95% CI: −1.50 to 2.54).

No significant difference was detected between the regional subgroups (*p* = 0.75) (Supplementary material 7).

#### 3.3.2 Leave-one-out sensitivity analysis

The pooled mean difference remained stable (range: 0.38–0.39) after sequentially removing each study, indicating that no single study had a disproportionate influence on the overall estimate. (Supplementary Material 8).

#### 3.3.3 Publication bias

Visual inspection of the funnel plot revealed a symmetrical distribution of the studies. Egger's test (*t* = 1.00, df = 20, *p* = 0.3291) and Begg's test (z = 0.14, *p* = 0.8879) revealed no evidence of publication bias. (Supplementary material 9).

### 3.4 PD versus multiple system atrophy

The pooled analysis revealed no significant difference in NLR between PD and MSA (MD = −0.30; 95% CI: −1.52 to 0.93; I^2^ = 0%) (Supplementary material 10).

### 3.5 Akinetic rigid versus tremor-dominant subtype

The difference in NLR between akinetic-rigid and tremor-dominant PD subtypes was also not significant (MD = 0.33; 95% CI: −0.62 to 1.27; I^2^ = 72.9%) (Supplementary material 11).

### 3.6 Correlation between the NLR and UPDRS I score

Two studies [35], [37] have evaluated the correlation between NLR and the UPDRS I subscore.

The pooled correlation using a random-effects model showed a moderate positive correlation (*r* = 0.42; 95% CI: 0.33 to 0.51) (Supplementary material 12).

### 3.7 Correlation between the NLR and UPDRS II scores

The pooled analysis from two studies [35], [37] indicated a weak to moderate positive correlation (*r* = 0.34; 95% CI: 0.24 to 0.44) for the UPDRS II subscore, which reflects activities of daily living (Supplementary material 13).

### 3.8 Correlation between the NLR and UPDRS III scores

**Fig. 3:**
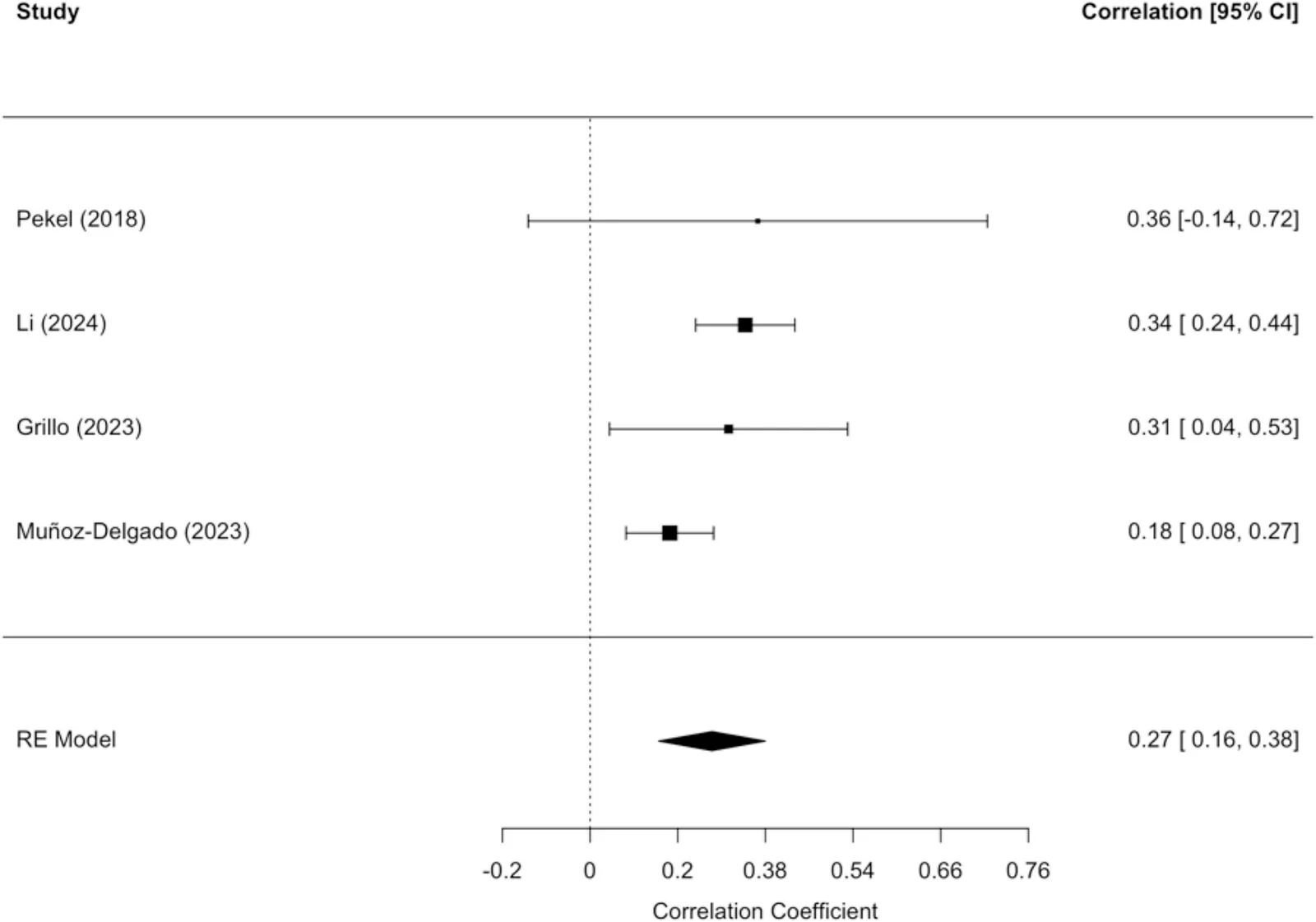
Correlation between NLR and UPDRS III.

Four studies [35], [37], [47], [50] assessed the correlation between NLR and UPDRS III scores in patients with PD. The correlation coefficients ranged from 0.18 (95% CI, 0.08 to 0.27) to 0.36 (95% CI, −0.14 to 0.72). The pooled correlation coefficient under the random-effects model was 0.27 (95% confidence interval [CI]: 0.16 to 0.38), indicating a positive association between NLR levels and motor symptom severity (Fig. 3).

### 3.9 Correlation between NLR and total UPDRS score

The overall UPDRS total score, representing global disease severity, was analyzed in two previous studies [35], [37]. Our meta-analysis revealed a moderate positive correlation (*r* = 0.67; 95% CI: 0.07–0.92), indicating that elevated NLR levels correlate with increased total UPDRS scores (Supplementary Material 14).

### 3.10 Correlation between the NLR and the Hoehn–Yahr score

Three studies [37], [45], [50] evaluated the correlation between the NLR and disease severity assessed by the Hoehn and Yahr (H&Y) scale in patients with PD. The pooled correlation coefficient obtained using a random-effects model was 0.33 (95% CI, 0.24–0.41), indicating a moderate positive correlation between NLR levels and H&Y stage (Supplementary material 15).

### 3.11 Constipation

A previous study [40] evaluated the association between NLR and constipation in patients with Parkinson's disease. The mean NLR was 2.7 (SD 1.3) in patients with constipation and 2.5 (SD 1.6) in those without. The mean difference was 0.2 (95% CI: −0.4 to 0.8), indicating no significant difference between the groups (Table 2).

**Table 2:** Narrative synthesis of the neutrophil-to-lymphocyte ratio in patients with Parkinson's disease.

Outcome	Study	Sample size	NLR mean (SD) in poor outcome	NLR mean (SD) in good outcome	Mean difference (95% CI)	NLR cut point	Sensitivity (%)	Specificity (%)
Severity								
Constipation	Umehara et al	123⁎	2.7 (1.3)	2.5 (1.6)	0.2 (−0.4;0.8)	–	–	–
Depression symptoms	Paul et al	465	3.38 (1.9)	2.78 (1.4)	0.6 (0.24;0.96)	–	–	–

Prognosis								
Mild Cognitive Impairment (Follow: 3.15 years)	Contaldi et al	58	2.20 (0.83)	2.78 (0.93)	−0.58 (−1.08; −0.08)	2.295	69.6% (49.13%; 84.4%)	67.8% (49.34%; 82.07%)

NLR: Neutrophil to lymphocyte ratio; SD: Standard deviation; CI: Confidence interval.

For mild cognitive impairment, the poor outcome column corresponds to patients who developed mild cognitive impairment during follow-up. Note that the NLR was lower in this group, and that the reported cut-off identifies mild cognitive impairment for values below the threshold.

⁎ For constipation, the sample corresponds to the 123 patients with available Wexner Constipation Score data within the study cohort.

### 3.12 Depression symptoms

Paul et al. [36] investigated the relationship between NLR and DS in a cohort of 465 patients. The mean NLR was 3.38 (SD 1.9) in patients with depression and 2.78 (SD 1.4) in those without depressive symptoms. The mean difference was 0.6 (95% CI: 0.24; 0.96), suggesting higher NLR values among those with depressive symptoms (Table 2).

### 3.13 Mild cognitive impairment

Contaldi et al. [52] analyzed NLR values according to cognitive status in 58 patients with PD followed for a mean of 3.15 years. Contrary to the direction observed for motor severity, patients who developed mild cognitive impairment had a lower mean NLR (2.20; SD 0.83) than those who remained cognitively unimpaired (2.78; SD 0.93), with a mean difference of −0.58 (95% CI: −1.08 to −0.08). Consistent with this inverse direction, the reported cut-off of 2.295 identifies mild cognitive impairment for values below, rather than above, the threshold, with a sensitivity of 69.6% (95% CI: 49.13 to 84.4) and a specificity of 67.8% (95% CI: 49.34 to 82.07) (Table 2).

### 3.14 Certainty of the evidence

Certainty was appraised by outcome using the GRADE (Table 1). For PD versus healthy controls, certainty was moderate, downgraded for inconsistency (I^2^ = 90%). For PD versus MSA, certainty was moderate due to imprecision with null confidence intervals. For motor subtypes (akinetic-rigid vs. tremor-dominant), certainty was low due to substantial inconsistency and imprecision (intervals included no effect). For severity outcomes, certainty was moderate for correlation with UPDRS I and II (imprecision due to not reaching our prespecified correlation threshold, with ρ ≈ 0.39), high for UPDRS III, and moderate for UPDRS total (wide interval). Certainty was moderate for correlation with Hoehn–Yahr due to imprecision against our prespecified correlation threshold. Constipation certainty was moderate among non-motor outcomes due to the interval crossing the null, while depressive symptoms rated high certainty. For mild cognitive impairment, both sensitivity and specificity were rated moderate, downgraded once for serious imprecision because the confidence intervals extend below our prespecified performance threshold of 70% and derive from a single study of 58 participants. We did not downgrade further for risk of bias or indirectness: the contributing study scored 8 stars on the NOS, was prospective, and assessed the target condition with validated criteria in the population of interest.

## 4 Discussion

This systematic review with meta-analysis (28 studies; 3593 participants in the main comparison) found that the NLR is higher in patients with Parkinson's disease than in healthy controls, with moderate certainty. Moreover, higher NLR correlated with worse clinical status: moderate correlations with UPDRS I and Hoehn–Yahr stage, weak-to-moderate correlations with activities of daily living and motor severity (UPDRS II and III), and the strongest association with the UPDRS total score. NLR did not differ between Parkinson's disease and multiple system atrophy or between akinetic-rigid and tremor-dominant phenotypes. Among the non-motor outcomes, NLR was higher in patients with depressive symptoms but was not associated with constipation. For mild cognitive impairment, the single longitudinal study available showed lower rather than higher NLR values in patients who developed impairment, with only modest discriminatory performance.

The only previous meta-analysis of the NLR in PD reported a comparable elevation relative to healthy controls but stopped at that comparison [27]. Our work extends it by pooling correlations with five validated severity measures, by separating diagnostic from prognostic use, by interrogating heterogeneity rather than merely reporting it, and by grading each outcome. Overall, our results shift the reading of the NLR from a candidate diagnostic marker towards a non-specific index of inflammatory burden whose main potential lies in severity stratification. Our finding of higher NLR in PD compared with healthy controls (MD = 0.38) aligns with recent meta-analyses that support a diagnostic association between NLR and PD, reinforcing the concept of a shared peripheral neuroinflammatory signature in PD and related markers such as the albumin-to-fibrinogen ratio and the lymphocyte-to-monocyte ratio [27], [58]. In physiopathologically related neurodegeneration, a meta-analysis of observational studies on cognitive impairment similarly reported elevated NLR, suggesting that NLR indexes systemic immune activation rather than a PD-specific phenotype, albeit with notable between-study heterogeneity [59].

The interpretation of our null contrast between PD and MSA deserves particular caution, and the recent literature on atypical parkinsonian syndromes clarifies why. In these disorders, where clinical overlap and the absence of accessible markers are the main obstacles, non-specific peripheral indices have been examined extensively, and the NLR is elevated in progressive supranuclear palsy (PSP) relative to controls [38], [60] and carries prognostic value in MSA without discriminating it diagnostically from PD [61]. Yet their capacity to discriminate between individual parkinsonian syndromes remains limited, which is consistent with our own finding. More specific analytes appear to behave differently: interleukin patterns differ across the clinical phenotypes of PSP [62], and transferrin and vascular cell adhesion molecule 1 have been proposed as candidate discriminators among tauopathic atypical parkinsonian syndromes [63]. Our absence of a difference between PD and MSA should therefore be read as a limitation of the NLR as a standalone parameter, rather than as evidence that the peripheral inflammatory signature of the two entities is identical; separating these syndromes immunologically will probably require cytokine or protein level profiling rather than ratios derived from the complete blood count.

A related question is how the NLR compares with the other indices derived from the same blood sample. Several of the included studies measured more than one concurrently: Li et al. reported that the NLR, the LMR and the NHR were all correlated with PD severity, with no ratio clearly superior [37], whereas Liu et al. found the NHR to be more informative than either the MHR or the NLR [23]. Outside PD, the PLR has been proposed alongside the NLR to reflect distinct neuroinflammatory patterns [24], and the NHR has been related to regional cerebral perfusion in corticobasal syndrome [25]. Two observations follow. First, all these indices share the same numerator and therefore the same dependence on the neutrophil compartment, so their estimates are not independent. Second, because the primary studies rarely report them within a common analytical model, a formal quantitative comparison was not feasible in our review, and we refrained from indirect comparisons across studies that would have been confounded by differences in populations and cut-offs. Direct, within-cohort comparison of these ratios, ideally against cytokine and neuroimaging measures, is the most informative next step.

A further perspective our review could not cover is the relationship of these indices with neuroimaging. In PSP, serum interleukin levels and non-specific peripheral inflammatory parameters have been correlated with magnetic resonance imaging measures of midbrain and peduncular atrophy, providing a structural anchor for markers that are otherwise purely haematological [64]. In PD, peripheral inflammation has been associated with dopaminergic denervation on DAT imaging [47] and with glymphatic system function [65]. None of the eligible studies reported paired imaging and NLR data in a form that could be pooled, which is itself an evidence gap: anchoring peripheral ratios to imaging endpoints would help establish whether the correlations we observed reflect the underlying neurodegenerative process or the systemic consequences of advanced disease.

Going further than diagnosis, our meta-analyses found positive association between NLR and severity on UPDRS I (*r* = 0.42), II (*r* = 0.34), III (*r* = 0.27), UPDRS total (*r* = 0.67) and Hoehn and Yahr stage (*r* = 0.33), consistent with the peripheral to central inflammatory model of progression (neutrophil activation, relative lymphopenia, inflammatory “spill-over”) [57], [66]. In line with this, single-cohort studies have reported higher NLR values in the rigid-akinetic subtype together with worse motor scores, which is consistent with our UPDRS III and Hoehn and Yahr signals; our pooled subtype comparison did not reach significance, and such reports are heterogeneous and design-dependent, so attributing stable phenotypic discrimination to the NLR alone remains premature [67], [68]. Outside PD, a meta-analysis in Alzheimer's disease found that higher NLR values accompanied worse global cognitive performance [69], and a population-based cohort related baseline NLR to incident dementia, supporting its prognostic relevance [70]. Comparable associations have been described between higher NLR values and greater neurological disability in multiple sclerosis [71], [72], and a clinical cohort reported shorter survival with higher NLR in amyotrophic lateral sclerosis [73]. Taken overall, these convergences paint a picture of a nonspecific yet informative index of inflammatory burden that is linked to severity and progression, but which study design, matching, confounding control and blood-sampling timing may drive residual differences.

The association we observed between the NLR and depressive symptoms, the outcome with the highest certainty in our review, is consistent with meta-analytic data in mood disorders and with clinical studies linking higher NLR to greater depressive symptom severity [74], [75], and has a plausible biological substrate. Peripheral pro-inflammatory cytokines activate indoleamine 2,3-dioxygenase, diverting tryptophan away from serotonin synthesis and towards the kynurenine pathway, and reduce the availability of tetrahydrobiopterin and thereby dopamine synthesis in the basal ganglia, an effect that maps onto the anhedonia and psychomotor slowing characteristic of depression in PD. Chronic inflammation also blunts glucocorticoid receptor sensitivity, perpetuating hypothalamic-pituitary-adrenal axis activation and, through cortisol driven demargination and lymphocyte redistribution, the very neutrophilia and lymphopenia that the NLR measures [76]. This last point matters for interpretation: the NLR may be simultaneously a marker of the inflammatory process contributing to depression and a downstream consequence of the stress response associated with it, and a cross-sectional design cannot distinguish between the two. The relevance of this pathway is not confined to PD, since in PSP the neutrophil-to-haemoglobin ratio differed between patients with and without depressive symptoms whereas the NLR did not, suggesting that the inflammatory correlates of mood disturbance in parkinsonian syndromes may be captured better by other ratios [26]. Evidence on constipation, by contrast, is scarce and discordant, and our null result rests on a single study; given that constipation in PD is driven largely by enteric neurodegeneration and by dopaminergic and anticholinergic medication, a systemic haematological index would not be expected to track it closely.

The direction of the association with mild cognitive impairment requires separate comment, because it runs opposite to every other finding in our review. In the only longitudinal study available, patients who developed mild cognitive impairment had lower, not higher, baseline NLR values, which is at odds both with the positive correlations we observed for motor severity and with external evidence linking higher NLR to worse cognitive performance in Alzheimer's disease [69] and to incident dementia in population-based cohorts [70]. Several explanations are possible and cannot be distinguished with the available data. The estimate derives from 58 patients and a threshold optimised within the same sample, so the estimate is imprecise and may not be stable, even though the study itself was methodologically sound. Alternatively, the inflammatory correlates of cognitive and motor progression in PD may genuinely dissociate, a possibility consistent with the observation that the lymphocyte count, rather than the ratio itself, carried much of the cognitive signal in that cohort. It is also possible that disease duration, dopaminergic treatment or comorbidity, none of which was modelled, confound the comparison. Whatever the explanation, this discordance argues against any clinical use of the NLR for cognitive prognostication and, more broadly, against assuming that a single peripheral ratio behaves uniformly across the clinical domains of PD.

The NLR is a low-cost, accessible index of systemic inflammation derived from routine complete blood counts and estimable at the bedside [77]. Our findings support its interpretation as an inflammatory signal that may complement clinical assessment rather than as a disease-specific biomarker [27]. Clinically, it could contribute to risk stratification: since higher values correlate with worse motor and non-motor status, baseline measurement might help identify patients at risk of more rapid functional decline, who would warrant closer monitoring, more thorough assessment of comorbidities and early multidisciplinary support, and it could form one component of a broader risk algorithm [37]. Its use for cognitive monitoring, by contrast, is not currently supported, for the reasons set out above.

The certainty of the evidence ranged from low to high across outcomes. Imprecision was the dominant reason for downgrading, affecting nine of the twelve outcomes assessed, and it stemmed from two distinct sources: confidence intervals crossing the null in the comparisons where we found no difference, and estimates falling short of our prespecified thresholds of clinical relevance in the correlation and accuracy outcomes. Inconsistency was the second reason, and downgraded the principal comparison and the motor subtype contrast. Only the correlation with UPDRS III and the association with depressive symptoms retained high certainty. This distribution matters for how the review should be read: the strength of each conclusion is anchored in the certainty rating rather than in statistical significance alone, and the outcomes with the weakest support are precisely those that would be needed to justify clinical use. Future studies should therefore report prespecified sample size calculations for each outcome assessed, since imprecision is a remediable limitation and its persistence across this literature is the main obstacle to firmer conclusions. This is particularly relevant for populations in which the evidence remains scarce: most included studies were conducted in European or Asian samples, despite known differences in PD frequency across ethnic groups [78] and persistently limited research involving ethnic minorities [79].

The evidence base has to be read critically, and its architecture constrains what can be concluded about disease progression. All but one of the included studies were cross-sectional or retrospective, so the correlations we report describe covariation at a single point in time and cannot establish whether the NLR rises as the disease advances within a given patient; disease duration was reported inconsistently and almost never modelled, even though it is the most obvious confounder of any association between an inflammatory index and a severity scale. Three further features deserve emphasis. First, the Newcastle-Ottawa Scale classified every included study as low risk of bias, with scores between 6 and 8; this uniformity more likely reflects the limited discrimination of the scale, which awards stars for the reporting of design features rather than for the adequacy of confounding control, than genuine methodological homogeneity. Second, control of confounding was generally rudimentary: most studies matched only for age and sex, and comorbidities known to influence the NLR, such as diabetes, cardiovascular disease, chronic kidney disease, active infection, smoking and immunomodulatory treatment, were variably reported and rarely adjusted for, so part of the difference we pooled may be attributable to case mix rather than to PD itself. Third, pre-analytical conditions were largely unstandardised: the timing of venepuncture relative to the circadian rhythm, fasting status, the dopaminergic medication state and the analyser used were seldom specified, and no study reported a prespecified NLR threshold derived from an independent sample.

Several limitations qualify our conclusions. At the level of the evidence, none of our estimates supports a causal interpretation. The pooled mean difference of 0.38 should be understood as a group-level contrast whose magnitude is small relative to the within-population variability of the NLR in healthy adults, with correspondingly extensive overlap between distributions and no implication for individual-level classification. Heterogeneity in the principal comparison was high (I^2^ = 90%) and was not fully explained by our prespecified subgroup analyses; the significant subgroup difference by study design, with a positive association confined to retrospective series, is compatible with selection and reporting effects, and although Egger and Begg tests were not significant, these tests have low power and cannot exclude small-study effects.

At the level of the review, several outcomes rest on very few studies. The pooled correlations with UPDRS I, UPDRS II and the UPDRS total score derive from two studies each, the comparisons with MSA and between motor subtypes from two studies each, and constipation, depressive symptoms and mild cognitive impairment from a single study each, so the corresponding between-study variance estimates are unstable and the confidence intervals should be read as indicative rather than definitive. The pooling of correlation coefficients assumes comparable adjustment across studies, which was not the case, and correlations were extracted as reported rather than recomputed from individual data. Our search included grey literature through Google Scholar, but may have missed studies indexed regionally; relevant literature published subsequently is discussed narratively where it informs interpretation, but did not contribute to the pooled estimates. Finally, our review was not designed to compare the NLR with other inflammatory or imaging markers, and the primary studies did not report them in a form that would have permitted such a comparison.

## 5 Conclusion

In patients with PD, the NLR is higher than in healthy controls (moderate certainty) and correlates positively with clinical severity across the UPDRS and the Hoehn and Yahr stage (moderate certainty, high for UPDRS III) and with depressive symptoms (high certainty). We found no differences between PD and MSA or between motor subtypes; given the non-specific nature of the index and the imprecision of these estimates, this indicates that the NLR is inadequate as a standalone discriminator rather than that the inflammatory profiles of these entities are equivalent. Evidence for mild cognitive impairment rests on a single small study and runs in the opposite direction, precluding clinical use. The NLR is therefore best regarded as an accessible marker of inflammatory burden that may complement clinical assessment in severity stratification, not as a diagnostic biomarker. Future work should priorities prospective cohorts with prespecified thresholds, direct comparison with other haematological, cytokine and neuroimaging markers, and recruitment in under-represented populations of South America and Africa.

## Transparency statement

The lead author, Frank Mayta-Tovalino, confirms that this manuscript provides an honest, accurate, and transparent account of the reported study. All significant aspects of the study have been included, and any deviations from the planned (and, if applicable, registered) study have been fully explained. AI was used for English grammar checking.

## CRediT authorship contribution statement

**Miguel Cabanillas-Lazo:** Writing – review & editing, Writing – original draft, Visualization, Supervision, Formal analysis, Data curation. **Alvaro Montes-Baldarrago:** Methodology, Investigation, Conceptualization. **Sandra Uriol-Alvino:** Investigation, Formal analysis, Data curation, Conceptualization. **Hans Baltazar-Ñahui:** Software, Methodology, Investigation, Formal analysis, Data curation, Conceptualization. **Natalia B. Pereda-Leon:** Writing – original draft, Methodology, Investigation, Conceptualization. **Harold Jimenez-Alvarez:** Writing – original draft, Investigation, Formal analysis, Data curation, Conceptualization. **Frank Mayta-Tovalino:** Writing – review & editing, Writing – original draft, Supervision, Software, Investigation, Conceptualization.

## Declaration of competing interest

The authors declare that they have no known competing financial interests or personal relationships that could have appeared to influence the work reported in this paper.

## Data Availability

The data that support the findings of this study are available from the corresponding author upon reasonable request.

## References

[1] Radhakrishnan D., Goyal V. (2018). Parkinson’s disease: a review. Neurol. India.

[2] Dorsey E.R., Elbaz A., Nichols E., Abbasi N., Abd-Allah F., Abdelalim A. (2018). Global, regional, and national burden of Parkinson’s disease, 1990–2016: a systematic analysis for the global burden of disease study 2016. Lancet Neurol..

[3] Ou Z., Pan J., Tang S., Duan D., Yu D., Nong H. (2021). Global trends in the incidence, prevalence, and years lived with disability of Parkinson’s disease in 204 countries/territories from 1990 to 2019. Front. Public Health.

[4] García-Ramos R., López Valdés E., Ballesteros L., Jesús S., Mir P. (2016). Informe de la Fundación del Cerebro sobre el impacto social de la enfermedad de Parkinson en España. Neurologia.

[5] Findley L.J. (2007). The economic impact of Parkinson’s disease. Parkinsonism Relat. Disord..

[6] Driver J.A., Kurth T., Buring J.E., Gaziano J.M., Logroscino G. (2008). Parkinson disease and risk of mortality: a prospective comorbidity-matched cohort study. Neurology.

[7] Vázquez-Vélez G.E., Zoghbi H.Y. (2021). Parkinson’s disease genetics and pathophysiology. Annu. Rev. Neurosci..

[8] Zaman V., Shields D.C., Shams R., Drasites K.P., Matzelle D., Haque A. (2021). Cellular and molecular pathophysiology in the progression of Parkinson’s disease. Metab. Brain Dis..

[9] Joshi N., Singh S. (2018). Updates on immunity and inflammation in Parkinson disease pathology. J. Neurosci. Res..

[10] Öberg M., Fabrik I., Fabrikova D., Zehetner N., Härtlova A. (2021). The role of innate immunity and inflammation in Parkinson’s disease. Scand. J. Immunol..

[11] Ferrari C.C., Tarelli R. (2011). Parkinson’s disease and systemic inflammation. Parkinsons Dis..

[12] Armstrong M.J., Okun M.S. (2020). Diagnosis and treatment of Parkinson disease: a review. JAMA.

[13] Postuma R.B., Berg D., Stern M., Poewe W., Olanow C.W., Oertel W. (2015). MDS clinical diagnostic criteria for Parkinson’s disease. Mov. Disord..

[14] Adler C.H., Beach T.G., Hentz J.G., Shill H.A., Caviness J.N., Driver-Dunckley E. (2014). Low clinical diagnostic accuracy of early vs advanced Parkinson disease. Neurology.

[15] Visanji N.P., Mollenhauer B., Beach T.G., Adler C.H., Coffey C.S., Kopil C.M. (2017). The systemic synuclein sampling study: toward a biomarker for Parkinson’s disease. Biomark. Med.

[16] Beach T.G., Adler C.H. (2018). Importance of low diagnostic accuracy for early Parkinson’s disease. Mov. Disord..

[17] Mollenhauer B., Zimmermann J., Sixel-Döring F., Focke N.K., Wicke T., Ebentheuer J. (2019). Baseline predictors for progression 4 years after Parkinson’s disease diagnosis in the de novo Parkinson cohort (DeNoPa). Mov. Disord..

[18] Zahorec R. (2001). Ratio of neutrophil to lymphocyte counts: rapid and simple parameter of systemic inflammation and stress in critically ill. Bratisl. Lek. Listy.

[19] Zahorec R. (2021). Neutrophil-to-lymphocyte ratio, past, present and future perspectives. Bratisl. Lek. Listy.

[20] Bhat T., Teli S., Rijal J., Bhat H., Raza M., Khoueiry G. (2013). Neutrophil to lymphocyte ratio and cardiovascular diseases: a review. Expert. Rev. Cardiovasc. Ther..

[21] Li W., Hou M., Ding Z., Liu X., Shao Y., Li X. (2021). Prognostic value of neutrophil-to-lymphocyte ratio in stroke: a systematic review and meta-analysis. Front. Neurol..

[22] Ghozy S., El-Qushayri A.E., Varney J., Kacimi S.E.O., Bahbah E.I., Morra M.E. (2022). The prognostic value of neutrophil-to-lymphocyte ratio in patients with traumatic brain injury: a systematic review. Front. Neurol..

[23] Liu Z., Fan Q., Wu S., Wan Y., Lei Y. (2021). Compared with the monocyte to high-density lipoprotein ratio (MHR) and the neutrophil to lymphocyte ratio (NLR), the neutrophil to high-density lipoprotein ratio (NHR) is more valuable for assessing the inflammatory process in Parkinson’s disease. Lipids Health Dis..

[24] Madetko N., Migda B., Alster P., Turski P., Koziorowski D., Friedman A. (2022). Platelet-to-lymphocyte ratio and neutrophil-to-lymphocyte ratio may reflect differences in PD and MSA-P neuroinflammation patterns. Neurol. Neurochir. Pol..

[25] Chunowski P., Migda B., Madetko-Alster N., Migda A., Kutyłowski M., Królicki L. (2024). The possible connection between neutrophil-to-high-density lipoprotein ratio and cerebral perfusion in clinically established corticobasal syndrome: a pilot study. Front. Neurol..

[26] Markiewicz M., Madetko-Alster N., Otto-Ślusarczyk D., Duszyńska-Wąs K., Migda B., Chunowski P. (2025). Possible significance of neutrophil-hemoglobin ratio in differentiating progressive supranuclear palsy from depression: a pilot study. Diseases.

[27] Hosseini S., Shafiabadi N., Khanzadeh M., Ghaedi A., Ghorbanzadeh R., Azarhomayoun A. (2023). Neutrophil to lymphocyte ratio in Parkinson’s disease: a systematic review and meta-analysis. BMC Neurol..

[28] Page M.J., McKenzie J.E., Bossuyt P.M., Boutron I., Hoffmann T.C., Mulrow C.D. (2021). The PRISMA 2020 statement: an updated guideline for reporting systematic reviews. BMJ.

[29] Wells G.A., Shea B., O’Connell D., Peterson J., Welch V., Losos M. (2014). https://www.ohri.ca/programs/clinical_epidemiology/oxford.asp.

[30] Zeng L., Brignardello-Petersen R., Hultcrantz M., Siemieniuk R.A.C., Santesso N., Traversy G. (2021). GRADE guidelines 32: GRADE offers guidance on choosing targets of GRADE certainty of evidence ratings. J. Clin. Epidemiol..

[31] Schünemann H.J., Mustafa R.A., Brozek J., Steingart K.R., Leeflang M., Murad M.H. (2020). GRADE guidelines: 21 part 1. Study design, risk of bias, and indirectness in rating the certainty across a body of evidence for test accuracy. J. Clin. Epidemiol..

[32] Zhang Z., Wang Y., Wang J., Cai Y., Liu P., Liu S. (2024). The role of peripheral inflammation-related biomarkers in distinguishing Parkinson’s disease. Parkinsonism Relat. Disord..

[33] Wang L.X., Liu C., Shao Y.Q., Jin H., Mao C.J., Chen J. (2022). Peripheral blood inflammatory cytokines are associated with rapid eye movement sleep behavior disorder in Parkinson’s disease. Neurosci. Lett..

[34] Sanjari Moghaddam H., Ghazi Sherbaf F., Mojtahed Zadeh M., Ashraf-Ganjouei A., Aarabi M.H. (2018). Association between peripheral inflammation and DATSCAN data of the striatal nuclei in different motor subtypes of Parkinson disease. Front. Neurol..

[35] Büyükkoyuncu Pekel N. (2018). Parkinson’s disease: is it actually an inflammatory disorder?. Turk. J. Geriatr..

[36] Paul K.C., Kusters C., Furlong M., Zhang K., Yu Y., Folle A.D. (2022). Immune system disruptions implicated in whole blood epigenome-wide association study of depression among Parkinson’s disease patients. Brain Behav. Immun. Health.

[37] Li F., Weng G., Zhou H., Zhang W., Deng B., Luo Y. (2024). The neutrophil-to-lymphocyte ratio, lymphocyte-to-monocyte ratio, and neutrophil-to-high-density-lipoprotein ratio are correlated with the severity of Parkinson’s disease. Front. Neurol..

[38] Inci I., Kusbeci O.Y., Eskut N. (2020). The neutrophil-to-lymphocyte ratio as a marker of peripheral inflammation in progressive supranuclear palsy: a retrospective study. Neurol. Sci..

[39] Jiang S., Wang Y., Gao H., Luo Q., Wang D., Li Y. (2019). Cell ratio differences in peripheral blood between early- and late-onset Parkinson’s disease: a case-control study. Biomed. Res. Int..

[40] Umehara T., Oka H., Nakahara A., Matsuno H., Murakami H. (2020). Differential leukocyte count is associated with clinical phenotype in Parkinson’s disease. J. Neurol. Sci..

[41] Yazar H., Yazar T. (2019). Serum inflammation biomarkers are associated with stages of Parkinson’s disease. Ann. Med. Res..

[42] Wang Y., Gao H., Jiang S., Luo Q., Han X., Xiong Y. (2021). Principal component analysis of routine blood test results with Parkinson’s disease: a case-control study. Exp. Gerontol..

[43] Stanca I.D., Criciotoiu O., Neamtu S.D., Vasile R.C., Berceanu-Bora N.M., Minca T.N. (2022). The analysis of blood inflammation markers as prognostic factors in Parkinson’s disease. Healthcare (Basel).

[44] Song L., Zhang S., Li H., Hansson O., Sonestedt E., Borné Y. (2022). Comparison of risk factors for Parkinson’s disease, coronary events and ischemic stroke. NPJ Parkinsons Dis..

[45] Solmaz V., Pekdaş Genç E., Aksoy D., Çevik B., Kurt S.G., Benli İ. (2018). Serum neutrophil-lymphocyte ratios, C-reactive protein and sedimentation levels in Parkinson’s disease. Cukurova Med. J..

[46] Muñoz-Delgado L., Macías-García D., Jesús S., Martín-Rodríguez J.F., Labrador-Espinosa M.Á., Jiménez-Jaraba M.V. (2021). Peripheral immune profile and neutrophil-to-lymphocyte ratio in Parkinson’s disease. Mov. Disord..

[47] Muñoz-Delgado L., Labrador-Espinosa M.Á., Macías-García D., Jesús S., Benítez Zamora B., Fernández-Rodríguez P. (2023). Peripheral inflammation is associated with dopaminergic degeneration in Parkinson’s disease. Mov. Disord..

[48] Kara S.P., Altunan B., Unal A. (2022). Investigation of the peripheral inflammation (neutrophil-lymphocyte ratio) in two neurodegenerative diseases of the central nervous system. Neurol. Sci..

[49] Jin H., Gu H.Y., Mao C.J., Chen J., Liu C.F. (2020). Association of inflammatory factors and aging in Parkinson’s disease. Neurosci. Lett..

[50] Grillo P., Sancesario G.M., Bovenzi R., Zenuni H., Bissacco J., Mascioli D. (2023). Neutrophil-to-lymphocyte ratio and lymphocyte count reflect alterations in central neurodegeneration-associated proteins and clinical severity in Parkinson disease patients. Parkinsonism Relat. Disord..

[51] Genç F., Erdal A. (2019). Parkinson hastalığında nötrofil/lenfosit oranlarının değerlendirilmesi. Pamukkale Med. J..

[52] Contaldi E., Magistrelli L., Cosentino M., Marino F., Comi C. (2022). Lymphocyte count and neutrophil-to-lymphocyte ratio are associated with mild cognitive impairment in Parkinson’s disease: a single-center longitudinal study. J. Clin. Med..

[53] Ataç Uçar C., Gökçe Çokal B., Ünal Artık H.A., İnan L.E., Yoldaş T.K. (2017). Comparison of neutrophil-lymphocyte ratio (NLR) in Parkinson’s disease subtypes. Neurol. Sci..

[54] Akıl E., Bulut A., Kaplan İ., Özdemir H.H., Arslan D., Aluçlu M.U. (2015). The increase of carcinoembryonic antigen (CEA), high-sensitivity C-reactive protein, and neutrophil/lymphocyte ratio in Parkinson’s disease. Neurol. Sci..

[55] Xing N., Dong Z., Wu Q., Kan P., Han Y., Cheng X. (2022). Identification and validation of key molecules associated with humoral immune modulation in Parkinson’s disease based on bioinformatics. Front. Immunol..

[56] Jiang L., Zhong Z., Huang J., Bian H., Huang W. (2022). Monocyte to high-density lipoprotein ratio has a high predictive value for the diagnosis of multiple system atrophy and the differentiation from Parkinson’s disease. Front. Aging Neurosci..

[57] Kim R., Kang N., Byun K., Park K., Jun J.S. (2023). Prognostic significance of peripheral neutrophils and lymphocytes in early untreated Parkinson’s disease: an 8-year follow-up study. J. Neurol. Neurosurg. Psychiatry.

[58] Li Y.M., Xu X.H., Ren L.N., Xu X.F., Dai Y.L., Yang R.R. (2024). The diagnostic value of neutrophil to lymphocyte ratio, albumin to fibrinogen ratio, and lymphocyte to monocyte ratio in Parkinson’s disease: a retrospective study. Front. Neurol..

[59] Hung K.C., Liu C.C., Wu J.Y., Ho C.N., Lin M.C., Hsing C.H. (2023). Association between the neutrophil-to-lymphocyte ratio and cognitive impairment: a meta-analysis of observational studies. Front. Endocrinol. (Lausanne).

[60] Muñoz-Delgado L., Luque-Ambrosiani A., Benítez Zamora B., Macías-García D., Jesús S., Adarmes-Gómez A. (2024). Peripheral immune profile and neutrophil-to-lymphocyte ratio in progressive supranuclear palsy: case-control study and meta-analysis. Eur. J. Neurol..

[61] Zhang L., Cao B., Hou Y., Wei Q., Ou R., Zhao B. (2022). High neutrophil-to-lymphocyte ratio predicts short survival in multiple system atrophy. NPJ Parkinsons Dis..

[62] Madetko-Alster N., Otto-Ślusarczyk D., Wiercińska-Drapało A., Koziorowski D., Szlufik S., Samborska-Ćwik J. (2023). Clinical phenotypes of progressive supranuclear palsy: the differences in interleukin patterns. Int. J. Mol. Sci..

[63] Madetko-Alster N., Otto-Ślusarczyk D., Struga M., Chunowski P., Alster P. (2025). Potential role of transferrin and vascular cell adhesion molecule 1 in differential diagnosis among patients with tauopathic atypical parkinsonian syndromes. Diagnostics (Basel).

[64] Alster P., Otto-Ślusarczyk D., Kutyłowski M., Migda B., Wiercińska-Drapało A., Jabłońska J. (2024). The associations between common neuroimaging parameters of progressive supranuclear palsy in magnetic resonance imaging and non-specific inflammatory factors: pilot study. Front. Immunol..

[65] Lin R., Cai G., Chen Y., Zheng J., Wang S., Xiao H. (2025). Association of glymphatic system function with peripheral inflammation and motor symptoms in Parkinson’s disease. NPJ Parkinsons Dis..

[66] Lucero J., Gurnani A., Weinberg J., Shih L.C. (2024). Neutrophil-to-lymphocyte ratio and longitudinal cognitive performance in Parkinson’s disease. Ann. Clin. Transl. Neurol..

[67] Alagoz A.N., Dagdas A., Bunul S.D., Erci G.C. (2025). The relationship between systemic inflammatory index and other inflammatory markers with clinical severity of the disease in patients with Parkinson’s disease. Biomedicines.

[68] Yi H., Liang X., Xu F., Li T., Yang X., Wei M. (2024). Association between neutrophil-to-lymphocyte ratio and motor subtypes in idiopathic Parkinson’s disease: a prospective observational study. BMC Neurol..

[69] Mohammadi A., Mohammadi M., Almasi-Dooghaee M., Mirmosayyeb O. (2024). Neutrophil to lymphocyte ratio in Alzheimer’s disease: a systematic review and meta-analysis. PLoS One.

[70] Ramos-Cejudo J., Johnson A.D., Beiser A., Seshadri S., Salinas J., Berger J.S. (2021). The neutrophil to lymphocyte ratio is associated with the risk of subsequent dementia in the Framingham heart study. Front. Aging Neurosci..

[71] Fahmi R.M., Ramadan B.M., Salah H., Elsaid A.F., Shehta N. (2021). Neutrophil-lymphocyte ratio as a marker for disability and activity in multiple sclerosis. Mult. Scler. Relat. Disord..

[72] Hemond C.C., Glanz B.I., Bakshi R., Chitnis T., Healy B.C. (2019). The neutrophil-to-lymphocyte and monocyte-to-lymphocyte ratios are independently associated with neurological disability and brain atrophy in multiple sclerosis. BMC Neurol..

[73] Choi S.J., Hong Y.H., Kim S.M., Shin J.Y., Suh Y.J., Sung J.J. (2020). High neutrophil-to-lymphocyte ratio predicts short survival duration in amyotrophic lateral sclerosis. Sci. Rep..

[74] Mazza M.G., Lucchi S., Tringali A.G.M., Rossetti A., Botti E.R., Clerici M. (2018). Neutrophil/lymphocyte ratio and platelet/lymphocyte ratio in mood disorders: a meta-analysis. Prog. Neuro-Psychopharmacol. Biol. Psychiatry.

[75] Aydin Sunbul E., Sunbul M., Yanartas O., Cengiz F., Bozbay M., Sari I. (2016). Increased neutrophil/lymphocyte ratio in patients with depression is correlated with the severity of depression and cardiovascular risk factors. Psychiatry Investig..

[76] Miller A.H., Raison C.L. (2016). The role of inflammation in depression: from evolutionary imperative to modern treatment target. Nat. Rev. Immunol..

[77] Forget P., Khalifa C., Defour J.P., Latinne D., Van Pel M.C., De Kock M. (2017). What is the normal value of the neutrophil-to-lymphocyte ratio?. BMC. Res. Notes.

[78] Wright Willis A., Evanoff B.A., Lian M., Criswell S.R., Racette B.A. (2010). Geographic and ethnic variation in Parkinson disease: a population-based study of US Medicare beneficiaries. Neuroepidemiology.

[79] Siddiqi S., Ortiz Z., Simard S., Li J., Lawrence K., Redmond M. (2025). Race and ethnicity matter! Moving Parkinson’s risk research towards diversity and inclusiveness. NPJ Parkinsons Dis..

