# Supplementary material for: The neutrophil-to-lymphocyte ratio in Parkinson's disease: a systematic review and meta-analysis of its association with differential diagnosis, severity, and prognosis
Source: Clin Park Relat Disord. 2026 Aug 15;15:100494. doi: 10.1016/j.prdoa.2026.100494 (PMC13546878; doi:10.1016/j.prdoa.2026.100494)
Supplement: Supplementary file 1 [file mmc1.docx]

## **The Neutrophil-to-Lymphocyte Ratio in Parkinson's Disease: A Systematic Review and Meta-analysis of its association with differential diagnosis, severity, and prognosis**

**Supplementary material 1. Search strategy**

| Database | Search strategy | Result |
| --- | --- | --- |
| Pubmed | #1 “Parkinson Disease”[MH] OR parkinson*[tiab] OR “Parkinson disease”[OT] OR “paralysis agitan*”[tiab] OR “paralysis agitan*”[OT]  #2: "neutrophil to lymphoc*" [all fields] OR "neutrophil-to-lymphoc*"[all fields] OR "neutrophil/lymphoc*"[all fields] OR "NLR" [all fields] OR "neutrophil lymphoc*" [all fields] OR "granulocyte lymphoc*" [all fields] OR "granulocyte to lymphoc*" [all fields] OR "granulocyteto-lymphoc*"[all fields] OR "granulocyte/lymphoc*" OR "GLR" [all fields]  #3: #1 AND #2 | 207 |
| Scopus | #1 TITLE-ABS-KEY(Parkinson* OR “paralysis agitans”)  #2 ALL ("neutrophil to lymphoc*" OR “neutrophil-to-lymphoc*” OR “neutrophil/lymphoc*” OR "NLR" OR "neutrophil lymphoc*" OR "granulocyte lymphoc*" OR “granulocyte to lymphoc*” OR “granulocyteto-lymphoc*” OR “granulocyte/lymphoc*” OR "GLR")  #5: #1 AND #2 | 449 |
| WOS | #1 KP=(Parkinson* OR “paralysis agitans”) OR AK=(Parkinson* OR “paralysis agitans”) OR TI=(Parkinson* OR “paralysis agitans”) OR TS=(Parkinson* OR “paralysis agitans”) OR AB=(Parkinson* OR “paralysis agitans”) OR TS=(Parkinson* OR “paralysis agitans”)  #2 ALL=("neutrophil to lymphoc*" OR “neutrophil-to-lymphoc*” OR “neutrophil/lymphoc*” OR "NLR" OR "neutrophil lymphoc*" OR "granulocyte lymphoc*" OR “granulocyte to lymphoc*” OR “granulocyteto-lymphoc*” OR “granulocyte/lymphoc*” OR "GLR")  #3: #1 AND #2 | 92 |
| Google Scholar | (Parkinson*) AND (“neutrophil lymphocyte ratio” OR nlr) | Only first 100 results |
| Embase | #1 ('parkinson disease'/exp OR 'lewy bodies of parkinson disease' OR 'lewy bodies of parkinson`s disease' OR 'lewy bodies of parkinsons disease' OR 'lewy body parkinson disease' OR 'lewy body parkinson`s disease' OR 'lewy body parkinsons disease' OR 'parkinson dementia complex' OR 'parkinson disease' OR 'parkinson`s disease' OR 'parkinsons disease' OR 'idiopathic parkinsonism' OR 'paralysis agitans' OR 'primary parkinsonism')  #2 ('neutrophil lymphocyte ratio'/exp OR 'nlr (lymphocyte)' OR 'neutrophil lymphocyte ratio' OR 'neutrophil to lymphocyte ratio' OR 'neutrophil/lymphocyte ratio')  #3: #1 AND #2 | 61 |

**Supplementary material 2. Excluded studies**

| **Title** | **Author** | **Reason for exclusion** |
| --- | --- | --- |
| Machine Learning Modelling for Prospective Parkinson’s Disease - the importance of inflammatory biomarkers and IGF1 - UKB study | Allwright, M. | Other exposure |
| Neutrophil-to-lymphocyte ratio (NLR) at boundaries of Progressive Supranuclear Palsy Syndrome (PSPS) and Corticobasal Syndrome (CBS) | Alster, P. | Other patient population |
| Neutrophil-to-lymphocyte ratio as a potential differential diagnostic marker for alzheimer’s disease, major depressive disorder, and parkinson’s disease | Baykan, H. | Other patient population |
| Biomarker of neuroinflammation in Parkinson’s disease | Liu, T. | Other patient population |
| The evaluation of neutrophil-lymphocyte ratio in idiopathic Parkinson's disease | Solmaz, V. | Unable to access the article |
| Association between plasma essential fatty acid levels and motor function in Parkinson's disease | Umehara, T. | Other exposure |
| Peripheral Inflammation Links with the Severity of Clinical Phenotype in Spinocerebellar Ataxia | Yaimeé V. | Other patient population |
| Possible inflammatory mechanisms and predictors of Parkinson’s disease patients with fatigue (Brief Review) | Haili W. | Other outcome |

**Supplementary material 3. Characteristics of included studies**

| Study | Country | Study  design | Main Inclusion criteria | Main exclusion criteria | Sample size | Subgroups in Parkinson | Age (years) | Sex (male %) | Control criteria | Outcome measure | Cutt-off value | Blood sample time | Follow up time | Funder |  |  |  |  |  |  |  |  |  |  |  |  |  |
| --- | --- | --- | --- | --- | --- | --- | --- | --- | --- | --- | --- | --- | --- | --- | --- | --- | --- | --- | --- | --- | --- | --- | --- | --- | --- | --- | --- |
| Akil, 2014 | Turkey | Case-control study | PD patients | Patients with systemic or neurologic disease. Recent surgery | 101 | NO | **Healthy control:**  65.9 ± 10.10*  **Parkinson’s disease:**  69.5 ± 9.3* | **Healthy control:**  26 (52%)  **Parkinson’s disease:**  29 (56.8%) | Healthy subjects | Diagnosis (According to the neurologist evaluation) and clinical prognosis (H&E) | 2.25 | Measured at baseline | NR | Dicle University DUBAP |  |  |  |  |  |  |  |  |  |  |  |  |  |
| Ataç, 2017 | Turkey | Case-control study | PD patients | Patients with neurological, systemic, autoimmune or infectious diseases | 106 | TDT, ART | **Healthy control:**  70.50 ± 7.86*  **Parkinson’s disease:**  72.74 ± 8.66 * | **Healthy control:**  19 (31*.*7%)  **Parkinson’s disease:**  22 (47.8%) | Healthy subjects | Clinical prognosis (UPDRS) | NR | Measured at baseline | NR | Self-funding |  |  |  |  |  |  |  |  |  |  |  |  |  |
| Contaldi, 2022 | Italy | Case-control study | PD patients | Patients with brain abnormalities, chronic, autoimmune or neurological diseases | 116 | PD-NC, PD-MCI | **Healthy control:**  69.31 ± 8.18 *  **Parkinson’s disease:**  69.28 ± 8.13 * | **Healthy control:**  42 (72.4%)  **Parkinson’s disease:**  42 (72.4%) | Healthy subjects | Diagnosis (According to the neurologist evaluation) and clinical prognosis (UPDRS and H&E) | 2.295 | Measured at baseline | NR | Department of Translational Medicine,  Piemonte Orientale University |  |  |  |  |  |  |  |  |  |  |  |  |  |
| Genç, 2019 | Turkey | Cohort study | PD patients | NR | 90 | TDT, ART | **Parkinson’s disease:**  67,31±9,2* | **Parkinson’s disease:**  60 (66,7%) | Neither | UPDRS, H&E (clinical prognosis) | 0.05 (Correlation coefficient) | Retrospectively obtained from the database system | 3 months | Self-funding |  |  |  |  |  |  |  |  |  |  |  |  |  |
| Grillo, 2023 | Italy | Case-control study | PD patients | Patients with neurodegenerative diseases  or under Immunosuppressant therapies | 121 | NR | **Healthy control:**  59.20 ±8.05  **Parkinson’s disease:**  59.78 ±9.11 | **Healthy control:**  34 (56.6%)  **Parkinson’s disease:**  42 (68.8%) | Healthy subjects | Clinical prognosis (MMSE, NMSS, UPDRS, H&E) | NR | Within 30 min from  lumbar puncture in a fasting state | NR | Self-funding |  |  |  |  |  |  |  |  |  |  |  |  |  |
| Hong, 2020 | China | Case-control study | PD patients | Systemic or neurological disease | 272 | EO, LO | **Healthy control:**  62.10 ± 7.13  **Parkinson’s disease:**  59.70 ± 10.58 | NR | Neither | Clinical prognosis (UPDRS, H&E, NMSQ, MOCA, MMSE) | 1.55 | Measured at baseline | NR | National Key R&D Program of  China |  |  |  |  |  |  |  |  |  |  |  |  |  |
| Kara, 2022 | Italy | Case-control study | PD patients | Systemic or neurological disease | 255 | NR | **Healthy control:**  62.10 ± 7.13*  **Parkinson’s disease:**  68.2 ± 9.3* | **Healthy control:**  31 (50.8%)  **Parkinson’s disease:**  49 (49%) | Healthy subjects | Diagnosis (According to the neurologist evaluation) and clinical prognosis (H&E) | 1.86 | Retrospectively obtained from the database system | NR | Self-funding |  |  |  |  |  |  |  |  |  |  |  |  |  |
| Liu, 2021 | China | Case-control study | PD patients | Neurodegenerative, endocrinological, immunologic or systemic disease. | 198 | NR | **Healthy control:**  67.5 (56.75 ~ 75) **  **Parkinson’s disease:**  68.5 (59 ~ 77) ** | **Healthy control:**  52 (53.1%)  **Parkinson’s disease:**  55 (56.1%) | Healthy subjects | Diagnosis (According to the neurologist evaluation) and clinical prognosis (H&E) | 2.62 | Retrospectively obtained from the database system | NR | Clinical Medical Research  Center Project of Qinghai Province |  |  |  |  |  |  |  |  |  |  |  |  |  |
| Madetko, 2022 | Poland | Case-control study | PD patients or Multiple System Atrophy patients | Age under 35, immunological or cardiovascular disease, drug use | 225 | NR | **Healthy control:**  59  **Parkinson’s disease:**  62.5  **MSA-P**: 62** | **Healthy control:**  41 (41.41%)  **Parkinson’s disease:**  55 (56.12%)  **MSA-P**:  10 (35.71%) | Healthy subjects | Diagnosis (According to the neurologist evaluation) | NR | Retrospectively obtained from the database system | NR | None |  |  |  |  |  |  |  |  |  |  |  |  |  |
| Muñoz, 2023 | Spain | Cohort study | PD patients | Patients with, systemic disease or immunosuppressive therapy | 555 | NR | **Primary cohort:** 56 ± 9.51  **PPMI replication cohort:**  61 ± 9.69 | **Primary cohort:** 134 (63.55 %)  **PPMI replication cohort:**  225 (65.41 %) | Neither | Clinical prognosis (UPDRS & MOCA) | NR | Measured at baseline | 6 months | Andalusian Public Health System Biobank and ISCIII-Red de Biobancos |  |  |  |  |  |  |  |  |  |  |  |  |  |
| Muñoz, 2021 | Spain | Case-control study | PD patients | Patients with systemic disease or immunosuppressive therapy | 732 | NR | **Healthy control:**  59.23 ± 15.06*  **Parkinson disease**:  63.7 ± 11.91* | **Healthy control:**  170 (47.89%)  **Parkinson’s disease:**  219 (58.09%) | Healthy subjects | Clinical prognosis (H&E) | NR | Retrospectively obtained from the database system | NR | Spanish Ministry of  Science and Innovation, Instituto de Salud  Carlos III-Fondo Europeo de Desarrollo Regional. |  |  |  |  |  |  |  |  |  |  |  |  |  |
| Solmaz, 2018 | Turkey | Case-control study | PD patients | Patients with systemic or neurological disease | 161 | NR | **Parkinson disease**:  66.43 ± 10.9  **Healthy control:**  63.34 ± 8.9 | **Parkinson disease**:  58 (57,4%)  **Healthy control:**  35 (59,4%) | Healthy subjects | Diagnosis (According to the neurologist evaluation) and clinical prognosis (H&E) | 2.25 | Measured at baseline | NR | Self-funding |  |  |  |  |  |  |  |  |  |  |  |  |  |
| Song, 2022 | China | Cohort study | Patients with incident disease status | Patients who developed both PD and coronary events or ischemic stroke | 26 210 | NR | **PD:**  60.9 ± 6.9*  **Coronary events:** 61.3 ± 7.0*  **Ischemic stroke:** 61.6 ± 7.1 * | **PD:**  250 (51.6%)  **Coronary events:**  1870 (56.9%)  **Ischemic stroke:**  1390 (46.8 %) | Neither | NR | NR | NR | 21.1 ± 6.1 years | Lund University |  |  |  |  |  |  |  |  |  |  |  |  |  |
| Stanca, 2022 | Romania | Case-control study | PD patients | Patients with systemic disease, abnormal brain CTs or drug use | 91 | NR | **Parkinson disease**:  65.91 ± 8.65*  **Healthy control:**  62.65 ± 13.39* | **Parkinson disease**:  22 (57.77%)  **Healthy control:**  23 (50%) | Healthy subjects | Clinical prognosis (UPDRS, H&E) | NR | 12 h after fasting between 8 am and 12 pm in the morning | NR | University of Medicine and Pharmacy  of Craiova |  |  |  |  |  |  |  |  |  |  |  |  |  |
| Wang, 2021 | China | Case-control study | PD patients | Patients with systemic or neurodegenerative disease | 889 | NR | **Parkinson disease**:  63*  **Healthy control:**  61* | **Parkinson disease**:  250 (55.19%)  **Control group:** 222 (50.92) | Subjects who matched PD patients based on age, sex and comorbidities | Clinical prognosis (H&E) | NR | Between 9 am and 11 am on the second day of hospitalization | NR | National Natural Science  Foundation of China |  |  |  |  |  |  |  |  |  |  |  |  |  |
| Yazar, 2019 | Turkey | Case-control study | PD patients | Patients with systemic disease,  abnormal cranial imaging findings, smoking  and alcohol habits, or  corticosteroid medication | 411 | NR | **Parkinson disease females:** 73.809±4.845  **Parkinson disease males:**  73.327±4.364  **Healthy control females:** 74.119±3.235  **Healthy control males:**  73.717±3.133 | **Parkinson disease:**  101 (48.09%)  **Healthy control:** 99 (49.5%) | Healthy subjects | Clinical prognosis (H&E, UPDRS, MMSE, GDRS) | NR | After 12 hours of fasting between 08:00 and 12:00 in the morning | NR | Self-funding |  |  |  |  |  |  |  |  |  |  |  |  |  |
| Umehara, 2021 | Japan | Cohort study | PD patients | Patients who were 40 years old or younger and those with  systemic disease or recent trauma | 142 | Akinetic rigid type, Tremor dominant type, Mixed type | NR | **PON:**  26 (64.9 ± 10.0)  **POL:**  11 (26.5 ± 9.3)  **POM:**  2 (5.8 ± 1.5)  **POE:**  1 (2.3 ± 2.1)  **POB:**  1 (0.5 ± 0.2) | Neither | Clinical prognosis (H&E, UPDRS, MMSE) | NR | In the morning after an overnight fasting | NR | Self-funding |  |  |  |  |  |  |  |  |  |  |  |  |  |
| Jiang, 2019 | China | Case-control study | PD patients | Patients with systemic disease or who use immunosuppressant or immune booster | 216 | Early-onset Parkinson’s disease and late-onset Parkinson’s disease | **EOPD:**  48.00 (7.00)  **E-Control:**  46.50 (5.00)  **LOPD:**  60.00 (6.00)  **L-Control:**  61.00 (9.00) | **EOPD:**  33 (53.2%)  **E-Control:**  16 (38.1%)  **LOPD:**  26 (47.3%)  **L-control**:  23 (40.4%) | Healthy subjects | Diagnosis (According to the neurologist evaluation) and clinical prognosis (H&E) | 4.53 | On the second day after admission | NR | National Natural Science Foundation of China |  |  |  |  |  |  |  |  |  |  |  |  |  |
| Zhang, 2024 | China | Case-control study | PD patients | Patients with systemic diseases or immunological alterations | 160 | NR | **Parkinson disease**:  64.35 ± 7.98*  **Healthy control**:  64.49 ± 7.98* | **Healthy control**: 32 (40%)  **Parkinson disease**:  32 (40%) | Healthy subjects | Diagnosis (According to the neurologist evaluation) and clinical prognosis (UPDRS, H&E, MOCA, MMSE, HAMA, AHRS, RBDSQ,Wexner) | NR | In the morning after 8 hours of fasting period | NR | National Science Foundation for Young Scientists of China, Tianjin Key Medical Discipline Construction Project,China and Tianjin Health Research Project,China |  |  |  |  |  |  |  |  |  |  |  |  |  |
| Li, 2024 | China | Case-control study | PD patients | Neurodegenerative, infectious, inflammatory diseases, liver, kidney or cerebral ischemic injuries | 606 | NR | **Parkinson disease**:  65.04 ± 10.40*  **Healthy control:**  63.78 ± 8.73* | **Parkinson disease**:  163 (53.8%)  **Healthy control**: 142 (46.8%) | Healthy subjects | Clinical prognosis (UPDRS, H&E, MOCA, MMSE) | 1.94 | In the morning after 8 hours of fasting period | NR | Initiated Foundation of Zhujiang Hospital and Science and Technology Program of Guangdong of China |  |  |  |  |  |  |  |  |  |  |  |  |  |
| Kim, 2023 | Korea | Cohort study | PD patients | Patients with cancer, hematological malignancy or taking immunosuppressive drugs | 554 | NR | **Parkinson disease:**  62.2 ± 6.08*  **Healthy control:** 61.5 ± 6.2* | **Parkinson disease**:  244 (65%)  **Healthy control**: 115 (65%) | Healthy subjects | Clinical prognosis (UPDRS, MOCA, MMSE) | NR | Measured at baseline and annually for 8 years | 8 years | Bio & Medical Technology Development Program of the National Research Foundation |  |  |  |  |  |  |  |  |  |  |  |  |  |
| Xing, 2022 | USA | Case-control study | PD patients | Other neurological disorder, acute or chronic inflammation or malignancy | 105 | NR | **Parkinson disease:**  65.96 ± 8.961  **Healthy control:** 68.75 ± 9.82 | **Parkinson disease**:  39 (56.5%)  **Healthy control**: 19 (52.7%) | Subjects who matched PD patients based on age and sex | NR | NR | Measured on the next day of hospitalization | NR | Tianjin  Municipal Science and Technology Bureau, China |  |  |  |  |  |  |  |  |  |  |  |  |  |
| Wang, 2022 | China | Case-control study | PD patients | PD dementia, acute infection or chronic disease | 189 | PD with REM sleep behavior disorder  PD with no REM sleep behavior disorder | **PD with REM sleep behavior disorder:**  65 (60 - 70)  **PD with no REM sleep behavior disorder:**  61 (51 - 68)  **Healthy control:** 61 (57 - 67) | **PD with REM sleep behavior disorder:**  37(60.2%)  **PD with no REM sleep behavior disorder:**  60(65%)  **Healthy control:** 19(52.8%) | Subjects who matched PD patients based on age and sex | Clinical prognosis (UPDRS, MOCA, MMSE) | NR | In the morning after an overnight fasting | NR | Suzhou Science and Technology  Development Program |  |  |  |  |  |  |  |  |  |  |  |  |  |
| Sanjari, 2018 | USA | Case-control study | PD patients | Patients who were not recruited for the Parkinson Progression Markers Initiative | 536 | Postural instability and gait difficulty  Indeterminate  Tremor dominant | **Postural instability and gait difficulty:**  60.8 (58.6–63.0)  **Indeterminate:**  62.8 (58.8–66.8)  **Tremor dominant:**  61.8 (60.7–62.9)  **Healthy control:**  61.1 (59.3–62.9) | **Postural instability and gait difficulty:**  44 (60.0%)  **Indeterminate:**  21 (77.7%)  **Tremor dominant:**  189 (65.6%)  **Healthy control:**  99 (66.9%) | Subjects who matched PD patients based on age and sex | Clinical prognosis (UPDRS, MOCA, SCOPA -AUT) | NR | Measured at baseline | NR | None |  |  |  |  |  |  |  |  |  |  |  |  |  |
| Pekel, 2018 | Turkey | Case-control study | PD patients | Patients with autoimmune, neoplastic, inflammatory or infectious diseases | 38 | NR | **Parkinson disease:**  65.06±6.38  **Healthy control:** 62.29±5.77 | **Parkinson disease**:  7 (41.2%)  **Healthy control**:  7 (33.3) | Subjects who matched PD patients based on age, sex and body mass index | Clinical prognosis (UPDRS, H&Y) | 2.25 | Measured at baseline | NR | None |  |  |  |  |  |  |  |  |  |  |  |  |  |
| Paul, 2022 | USA | Case-control study | PD patients | Patients who were not recruited for the Parkinson’s disease, Environment and Genes study | 692 | PD with No  Depression  History  PD with Lower Depressive  Symptoms  PD with Higher Depressive  Symptoms | **PD with No**  **Depression**  **History:**  71.8 (9.4)  **PD with Depression History:**  68.8 (10.0)  **PD with Lower Depressive**  **Symptoms**  71.2 (9.43)  **PD with Higher Depressive**  **Symptoms**  70.2 (10.3)  **Healthy control with No**  **Depressive Symptoms**  69.4 (12.0)  **Healthy control with Depression**  **History**  62.1 (13.0)  **Healthy control with Higher Depressive**  **Symptoms:**  62.1 (13.0) | **PD with No**  **Depression**  **History:**  211 (63%)  **PD with Depression History:**  71 (55%)  **PD with Lower Depressive**  **Symptoms**  195 (58%)  **PD with Higher Depressive**  **Symptoms**  86 (66%)  **Healthy control with No**  **Depressive Symptoms**  102 (61%)  **Healthy control with Depression**  **History**  35 (51%)  **Healthy control with Higher Depressive**  **Symptoms:**  11 (48%) | NR | Clinical prognosis (UPDRS, H&Y) | 2.3-3.0 | Measured at baseline | NR | National Institute on Aging and National Institute of Environmental Health Sciences |  |  |  |  |  |  |  |  |  |  |  |  |  |
| Jiang, 2022 | China | Case-control study | PD patients | Neurodegenerative, autoimmune, infectious diseases | 296 | NR | **Parkinson disease:**  63 (56–68)  **Multiple system atrophy:**  61 (55–66)  **Healthy control:** 58 (52–67.75) | **Parkinson disease:**  72 (57.6%)  **Multiple system atrophy:**  30 (63.8%)  **Healthy control:** 60 (48.4%) | Subjects who matched PD patients based on age and sex | Diagnosis | 2.99 | Measured at baseline | NR | None |  |  |  |  |  |  |  |  |  |  |  |  |  |
| Inci, 2019 | Turkey | Case-control study | Progressive supranuclear palsy and PD patients | History of trauma and surgery 3 months before, smoking or obese | 103 | NR | **Parkinson disease:**  66.59 ± 9.54 **Progressive supranuclear palsy:**  68.28 ± 8.7 **Healthy control:** 65.05 ± 6.52 | **Parkinson disease:**  23 (54.76%)  **Progressive supranuclear palsy:**  17 (80.95%)  **Healthy control:** 22 (55%) | Subjects who matched patients based on age | Diagnosis | 2.34 | Measured at baseline | NR | None |  |  |  |  |  |  |  |  |  |  |  |  |  |
| **MSA-P: Multiple system atrophy parkinsonian type**  **TDT: Tremor-dominant typer ART: Akinetic-rigid type**  **MXT: Mixed type**  **EO: Early onset**  **LO: Late onset**  **UPDRS: Unified Parkinson's Disease Rating Scale**  **ACE-R: Addenbrooke’s Cognitive Examination Revised**  **MMSE: Mini Mental State Examination**  **MOCA: Montreal Cognitive Assessment**  **H&E: Hoehn y Yahr scale**  **NMSS: Non-Motor Symptoms Scale**  **NMSQ: Non-Motor Symptoms Questionnaire**  **HAMA: Hamilton Anxiety Scale**  **AHRS: Argentina Hyposmia Rating Scale**  **RBDSQ: Rapid Eye Movement Sleep Behavior Disorder Screening Questionnaire**  **Wexner: Wexner Constipation Score**  **NR: No reported**  **PD-NC: PD-normal cognition**  **PD-MCI: PD with**  **mild cognitive impairment**  **MSA-P: Multiple System Atrophy patients**  **SCOPA-AUT: Scales for Outcomes in Parkinson’s disease.**  *** Mean and standard deviation**  **** Median and range** | | | | | | | | | | | | | |  |  |  |  |  |  |  |  |  |  |  |  |  |  |

**Supplementary material 4. Newcastle-Ottawa Scale (NOS) assessment.**

| Study (Cohort) | Selection (max 4*) | | | | Comparability (max 2*) | Exposure (max 3*) | | |  |  |
| --- | --- | --- | --- | --- | --- | --- | --- | --- | --- | --- |
|  | **Representativeness of the exposed cohort** | **Selection of the non exposed cohort** | **Ascertainment of exposure** | **Definition of cohorts** | **Comparability of cohorts on the basis of the design or analysis** | **Assessment of outcome** | **Was follow-up long enough for outcomes to occur** | **Adequacy of follow up of cohorts** | **Score** | **Risk of bias** |
| Muñoz  (2023) | * | * | * | * | ** | * |  | * | 8 | Low risk of bias |
| Solmaz  (2018) | * | * | * |  | * | * |  | * | 6 | Low risk of bias |
| Song  (2022) | * | * | * | * | * | * | * | * | 8 | Low risk of bias |
| Stanca  (2022) | * | * | * |  | ** | * |  | * | 7 | Low risk of bias |
| Yazar  (2019) | * | * | * | * | * | * |  | * | 7 | Low risk of bias |
| Genç, (2019) | * | * | * |  | * | * |  | * | 6 | Low risk of bias |
| Umehara (2021) | * | * | * | * | ** | * |  | * | 8 | Low risk of bias |

| Study (Case control) | Selection (max 4*) | | | | Comparability (max 2*) | Exposure (max 3*) | | |  |  |
| --- | --- | --- | --- | --- | --- | --- | --- | --- | --- | --- |
|  | **Is the case definition adequate?** | **Representativeness of the cases** | **Selection of controls** | **Definition of controls** | **Comparability of cases and controls on the basis of the design or analysis** | **Ascertainment of exposure** | **Same method of ascertainment for cases and controls** | **Non-Response rate** | **Score** | **Risk of bias** |
| Muñoz  (2021) | * | * | * | * | ** | * | * | * | 9 | Low risk of bias |
| Muñoz  (2023) | * | * | * |  | ** | * | * | * | 8 | Low risk of bias |
| Solmaz  (2018) | * | * | * |  | * | * | * | * | 7 | Low risk of bias |
| Stanca  (2022) | * | * | * | * | * | * | * | * | 8 | Low risk of bias |
| Wang  (2021) | * | * | * | * | ** | * | * | * | 9 | Low risk of bias |
| Yazar  (2019) | * | * | * | * | * | * | * | * | 8 | Low risk of bias |
| Jiang  (2019) | * | * | * | * | ** | * | * | * | 9 | Low risk of bias |
| Zhang  (2024) | * | * | * |  | ** | * | * | * | 8 | Low risk of bias |
| Akil  (2014) | * | * | * | * | * | * | * | * | 8 | Low risk of bias |
| Ataç, (2017) | * | * | * | * | * | * | * | * | 8 | Low risk of bias |
| Contaldi (2022) | * | * |  |  | * | * | * | * | 6 | Low risk of bias |
| Grillo (2023) | * | * | * | * | * | * | * | * | 8 | Low risk of bias |
| Hong (2020) | * | * | * | * | * | * | * | * | 8 | Low risk of bias |
| Kara (2022) | * | * | * | * | ** | * | * | * | 9 | Low risk of bias |
| Liu (2021) |  | * | * | * | ** | * | * | * | 8 | Low risk of bias |
| Madetko (2022) | * | * | * | * | * | * | * | * | 8 | Low risk of bias |
| Li (2024) | * | * |  |  | * | * | * | * | 6 | Low risk of bias |
| Kim (2023) | * | * | * |  | * | * | * | * | 7 | Low risk of bias |
| Xing  (2022) |  |  | * |  | * | * | * | * | 5 | Moderate risk of bias |
| Wang  (2021) | * | * | * |  | * | * | * | * | 7 | Low risk of bias |
| Sanjari  (2021) | * | * | * | * | * | * | * | * | 8 | Low risk of bias |
| Pekel  (2021) | * | * | * | * | * | * | * | * | 8 | Low risk of bias |
| Paul  (2022) | * | * | * | * | * | * | * | * | 8 | Low risk of bias |
| Jiang  (2022) | * | * | * | * | ** | * | * | * | 9 | Low risk of bias |
| Inci  (2019) | * | * | * | * | * |  |  | * | 6 | Low risk of bias |

**Supplementary material 5. Subgroup analysis by study design**

**Supplementary material 6. Subgroup analysis by match case control.**

**Supplementary material 7. Subgroup analysis by geographic region**

**Supplementary material 8. Subgroup and sensitivity analysis (leave-one-out)**

**Supplementary material 9. Funnel plot for publication bias**

**Supplementary material 10. Forest plot of the mean difference between Parkinson Disease and Multiple System Atrophy**

**Supplementary material 11. Forest plot of the mean difference between Akinetic-rigid and tremor-dominant subtype**

**Supplementary material 12. Correlation between NLR and UPDRS I**

**Supplementary material 13. Correlation between NLR and UPDRS II**

**Supplementary material 14. Correlation between NLR and UPDRS Total**

**Supplementary material 15. Correlation between NLR and Hoehn and Yahr scale**
